# Transmission of Severe Acute Respiratory Syndrome

**DOI:** 10.3201/eid0909.030471

**Published:** 2003-09

**Authors:** Isao Arita, Kazunobu Kojima, Miyuki Nakane

**Affiliations:** * Agency for Cooperation in International Health, Kumamoto, Japan; † Sapporo Medical University School of Medicine, Sapporo, Japan

**Keywords:** severe acute respiratory syndrome (SARS), outbreak, transmission, close contact, laboratory diagnosis, clinical virology, international research cooperation

**To the Editor:** The worldwide pattern of severe acute respiratory syndrome (SARS) transmission in 2003 suggests that transmission has occurred more frequently in communities that share certain social and cultural characteristics. Of 8,500 probable cases since March, >90% were reported from China (including mainland, Hong Kong, and Macau) and Taiwan. Of the other 27 countries reporting SARS occurrences, 23 reported <10 cases and the others 1–3 cases. The small number of transmissions in these other countries suggests that the close contact required for transmission did not occur, whereas in China, community-based transmission has continued. In contrast, the relatively large number of cases in Canada, the United States, Singapore, and Vietnam (which comprise 7% to 10% of the total SARS cases worldwide) is related to the fact that relatively prolonged contact occurred because of the patients’ close cultural ties with China. Why does Japan have still no cases of SARS, despite its geographic proximity to the most affected areas? We suggest that transmission has not occurred because Japan remains a society mostly closed to non-Japanese persons and has a history of casual contact between its citizens and the travelers and noncitizens who reside there.

Hospitals have functioned as junctions for varied communities in spreading the SARS virus further. Because of SARS’ likely place of origin, the initial “community” included Chinese persons who then kindled the chain of transmission to other communities throughout the world. Daily, close contact between SARS patients and hospital personnel led to an unusually large number of infections among medical staff members. Effective prevention measures such as vaccines are not available and may be a factor in the spread of the infection.

Even in the era of globalization and mass air transit, most persons live inside a relatively small circle of community, made up of others of similar ethnicity, religious beliefs, educational level, and social class who live in the same vicinity; this sort of small circle has been described as “mutual coexistence” by anthropologist Kinji Imanishi (*1*). Basically, the SARS-associated coronavirus began circulating among members of such a community. This theory does not suggest that certain ethnic groups are predisposed to be susceptible to SARS.

Why have few cases of SARS occurred in children? All age groups are susceptible to the SARS virus, which is new to humans. However, adults have more chance to become infected through contacts in their daily lives, whereas children do not. Rapid isolation of the adult patients contributed to reduced frequency of exposure for children in that household, which is in contrast to the usual infectious diseases of childhood (since children do not have immunity against many age-old microbes).

Some contradictions exist for our interpretation of the SARS transmission pattern. Investigations have shown that in Canada, Hong Kong and elsewhere, some casual brief contact caused the infection or that the link between the source and the case was not at all clear. We may have missed other important routes of transmission, or a totally unknown element may be involved. Without an answer for this discrepancy, we note that the clinical virology for SARS, such as pattern of virus shedding and host immune response, is still developing (*2*). For example, a total of 19 cases in China were identified as SARS by coronavirus isolation, polymerase chain reaction, or serologic tests. For two case-patients, the results of three tests were positive; 10 case-patients had negative test results; and in 14 case-patients, the virus was not isolated. Interpreting these results is difficult. In the United States, 97% of the probable cases were attributed to a recent history of international travel to SARS-affected areas. Antibodies to SARS-associated coronavirus were demonstrated for 8 of 41 probable case-patients in convalescent-phase serum, bringing the proportion of laboratory-confirmed cases to 20%, even in the probable cases, and 0% among the suspected cases in the United States so far (*3*). These results are the best available by laboratories with the current limited technical knowledge. We are not persuaded that casual contact with SARS patients in unfamiliar settings results in contracting the disease.

The winter of 2003 will be critical for observing how the virus behaves, whether the winter climate accelerates the transmission, and how we handle that acceleration. Despite current global efforts, thin lines of transmissions may remain in China; the virus may flare up again. Officials in China and sites of the outbreak must interrupt as many chains of transmission as possible before October. Surveillance should also be intensified. Ongoing study to improve laboratory diagnosis and clinical virology is key, so that effective isolation can be practiced; at present, these measures are the only ones known to interrupt the transmission of SARS. The group on which to focus should be the community in close contact with previous outbreak areas.
